# Immunotherapy-Induced Overlap Syndrome: Myositis, Myasthenia Gravis, and Myocarditis—A Case Series

**DOI:** 10.1155/2024/5399073

**Published:** 2024-03-29

**Authors:** Nikhil Aggarwal, Diletta Bianchini, Rosemeen Parkar, Jennifer Turner

**Affiliations:** Kent Oncology Centre, Maidstone General Hospital, Maidstone and Tunbridge Wells NHS Trust, Maidstone, Kent, UK

## Abstract

Immune checkpoint inhibitors (ICI) are monoclonal antibodies that target immune checkpoint inhibitory receptors. They have revolutionised cancer treatment but can be associated with a wide range of adverse side effects. Rarely, they can be associated with the triad of myositis, myasthenia gravis, and myocarditis or overlap syndrome. Prompt recognition and early intervention are needed to treat these potentially life-threatening conditions. We report a case series of patients with ICI-related overlap syndrome, including the first with avelumab, and discuss the current management guidelines.

## 1. Introduction

Immunotherapy has revolutionised cancer treatment. Immune checkpoint inhibitors (ICIs) are monoclonal antibodies that target immune checkpoint inhibitory receptors such as cytotoxic T‐lymphocyte–associated protein 4 (CTLA‐4), programmed cell death receptor 1 (PD‐1), and programmed cell death ligand 1 (PD‐L1) [1]. These have become standard of care in multiple cancer types as single-agent therapy, dual checkpoint inhibition, and in combination with chemotherapy or targeted therapy [2]. These agents can, however, be associated with a wide range of life-threatening inflammatory and auto-immune-related adverse events (irAEs) affecting all organs, usually occurring within the first 2 months [3]. IrAEs are graded according to the Common Terminology Criteria for Adverse Events (CTCAE) [4].

Myocarditis is the most common and fatal cardiotoxicity, leading to death in 25–50% of cases [5]. Myasthenia gravis (MG) is the most commonly reported neuromuscular irAE, with 40–50% requiring ventilatory support, seven times higher than in typical MG [3]. ICI-related myositis is an inflammatory process affecting the skeletal muscles and has a poorer prognosis than spontaneous autoimmune myopathies [6]. Although myocarditis, myositis, and MG can occur in isolation, they sometimes occur together or in a triad, often referred to as overlap or triple “*m*” syndrome [2]. There is a need for increased awareness to suspect and treat these conditions. Here, we present four cases of patients with ICI-related overlap syndrome and to our knowledge the first resulting from avelumab. Written informed consent was gained from all patients or their next of kin.

## 2. Case Series

### 2.1. Patient 1

A 58-year-old male with a background of 1.91 mm melanoma, excised six years previously from his upper back, was referred urgently to oncology with a new biopsy-proven metastatic melanoma in his axilla.

His past medical history includes a vestibular tumour treated with stereotactic radiotherapy, hypertension, and depression. His Eastern Cooperative Oncology Group (ECOG) performance status [7] was zero, and he was a nonsmoker and led an active lifestyle.

A computerised tomography chest, abdomen, and pelvis (CT CAP) scan confirmed left supraclavicular and large left axillary lymph nodes, nodules adjacent to pleura in both lower lobes as well as an additional left lower pulmonary nodule.

The patient commenced treatment with 350 mg ipilimumab and 110 mg nivolumab. He was admitted to the hospital 10 days following the first cycle of treatment with palpitations, chest pain, and diplopia. An electrocardiogram (ECG) revealed left axis deviation and right bundle branch block. His blood results showed a creatine kinase (CK) 11,150 IU/L (normal range 40–320 IU/L), troponin-T 1051 ng/L (normal range <14 ng/L), ALT 415 U/L (normal range <41 U/L), and bilirubin 11 umol/L (normal range 0–29 umol/L). He was commenced on 100 mg once daily (OD) of intravenous (IV) methylprednisolone for probable ICI-related overlap syndrome and hepatitis. Twenty-four hours later, the patient developed a complete heart block and required temporary pacing and insertion of an externalised permanent single lead pacemaker. Mycophenolate mofetil (MMF) 1000 mg twice daily (BD) was added, and methylprednisolone was increased to 1000 mg OD due to worsening troponin-T (Table 1). The patient was transferred to a specialist cardio-oncology centre. Coronary angiogram revealed no flow-limiting lesions; however, endomyocardial biopsies revealed several small foci of lymphocytic inflammation. The patient's myocarditis, myositis, and hepatitis improved with immunosuppression; however, his diplopia worsened and he developed type 2 respiratory failure, requiring noninvasive ventilation, as well as bulbar symptoms including difficulty swallowing, requiring nasogastric (NG) feeding. Symptoms did not respond to methylprednisolone and intravenous immunoglobulins (IVIG) but improved with plasmapheresis and definitively with rituximab. Following a three-month stay in the hospital, including one month in the ICU, the patient was discharged with neurology, cardiology, and oncology follow-up.

### 2.2. Patient 2

A 78-year-old gentleman with a background of grade 3 metastatic bladder cancer was referred to oncology. His latest CT urogram and CT CAP revealed a large bladder tumour arising from the right lateral wall with extravesical extension and multiple enlarged retroperitoneal and right pelvic side wall lymph nodes.

The patient had no significant past medical history. He had a BMI of 42 and an ECOG performance status [7] of one. He was a retired pension fund investment manager and nonsmoker.

The patient was commenced on palliative chemotherapy with a combination of gemcitabine and carboplatin for six cycles. Staging CT scans throughout treatment showed favourable responses with no evidence of disease progression. The patient was hence commenced on maintenance immunotherapy with avelumab.

Following two cycles of 800 mg avelumab, each two weeks apart, the patient was admitted to the emergency department (ED) with extreme fatigue, unable to hold his head up after two minutes, and diplopia. His blood results revealed a CK > 8500 IU/L (normal range 40–320 IU/L) and troponin-T > 700 ng/L (normal range <14 ng/L). ECG showed normal sinus rhythm. To aid a diagnosis of myocarditis, troponin-I was also tested with a result of 151 ng/L (normal range 0–34). The patient was deemed to have ICI-induced myositis, myocarditis, and MG (later confirmed with positive antiacetylcholine receptor (anti-AChR) antibodies (0.47 nmol/L, normal range 0–0.25 nmol/L)). The patient was cautiously given 1000 mg IV methylprednisolone OD, being careful to monitor for decompensated MG crisis, to prevent a potentially fatal myocarditis. Pyridostigmine was initially avoided due to coexisting myocarditis and the risk of conduction defects. The patient developed tachypnoea with a respiratory rate of 40 per minute and forced vital capacity of 1.06 L (normal range 3–5 L). The patient was transferred to the intensive care unit (ICU), and IVIG was commenced. He was also commenced on pyridostigmine at a reduced dose of 30 mg three times per day (TDS) to lessen the risk of requiring intubation from MG. MMF 500 mg TDS was commenced due to a rapid rise of troponin-T to 1038 ng/L despite immunosuppression. The patient's symptoms improved, and he was successfully weaned from noninvasive ventilation and stepped down to the ward. He made a good recovery and was discharged with neurology, cardiology, and oncology follow-up, six weeks after first being admitted to the hospital.

### 2.3. Patient 3

A 77-year-old gentleman with a background of grade 3 T4N0M0 left clear cell renal cell carcinoma was referred to oncology following a CT CAP confirming disease progression in the peritoneal nodules and lung metastases. He had a past medical history of chronic pancreatitis and hypertension. His ECOG performance status [7] was zero and was a nonsmoker. The patient was commenced on first line 50 mg OD sunitinib, an oral TKI (tyrosine kinase inhibitor) achieving an excellent partial response. However, a CT CAP two years later revealed pancreatic metastases, new thoracic lymphadenopathy, and an increase in pulmonary metastases, right pleural, and peritoneal disease. The patient was, therefore, commenced on a second line 480 mg nivolumab.

Following two cycles of nivolumab, the patient developed dysphagia, dysphonia, and ptosis. CTs neck and head did not reveal any obvious morphological cause. Following review from the neurology team, the patient was deemed to have MG, although notably anti-AChR antibodies were negative. An NG was inserted due to the patient having an unsafe swallow. The patient also developed chest pain with ECG showing dynamic anterolateral *T*-wave inversion and ST depression with a significant troponin-T rise to 1786 ng/L (normal range <14 ng/L). It was felt that this could represent a lateral NSTEMI or myocarditis secondary to nivolumab. CK was 3217 IU/L (normal range 40–320 IU/L). The patient was diagnosed with overlap syndrome and commenced on IVIG, 130 mg IV (2 mg/kg) methylprednisolone OD, and 60 mg pyridostigmine six times per day. An angiogram was deemed not appropriate due to the increased risk of coronary vasospasm-induced ischaemia from possible myocarditis. The patient developed respiratory failure, appeared fatigued, and was cachexic. It was discussed with his next of kin that escalation of care with intubation and ventilation would not be in the patient's best interests. The patient deteriorated from a respiratory point of view despite a high-flow oxygen and subsequently passed away, three weeks after initially being admitted to hospital.

### 2.4. Patient 4

An 86-year-old gentleman with a background of pT4aN0N0 mucosal melanoma was referred to oncology for adjuvant radiotherapy following a medial maxillectomy. He had a past medical history of hypertension, type 2 diabetes mellitus, osteoporosis, cervical spondylosis, prostate cancer on leuprorelin, and a myocardial infarction 24 years previously. He was of ECOG performance status [7] zero and a nonsmoker.

**Figure 1:**
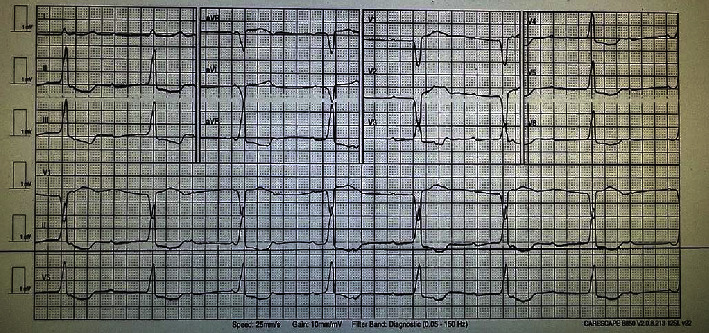
ECG of patient 4 showing complete heart block and left bundle branch block.

The patient underwent radiotherapy treatment 55 Gray in 20 fractions over 4 weeks to his nose. A nuclear medicine whole-body positron emission tomography (PET) CT three-month postradiotherapy revealed an interval left adrenal metastatic deposit and a small deposit in the left anterior chest wall. Following discussions in the multidisciplinary team meeting, the patient was deemed not to be a candidate for surgery due to comorbidities. The patient would not be a candidate for stereotactic ablative body radiotherapy (SABR) because of the short interval in which metastatic disease developed with likely micrometastatic disease. The patient was, therefore, commenced on first line palliative 200 mg pembrolizumab. He was admitted to the ED two weeks after his first cycle with increasing tiredness, diplopia, and slurred speech. His blood results revealed CK 6185 IU/L (normal range 40–320 IU/L), troponin-T 6188 ng/L (normal range <14 ng/L), ALT 882 U/L (normal range <41 U/L), and bilirubin 8 umol/L (normal range 0–29 umol/L). The patient was deemed to have ICI-related MG (later confirmed with positive anti-AChR antibodies (6.48 nmol/L, normal range 0–0.25 nmol/L)), myocarditis, myositis, and hepatitis. He was commenced on IV methylprednisolone 100 mg (2 mg/kg) OD. The following day, he developed a complete atrioventricular (AV) block on his ECG with left bundle branch block and some long pauses of >10 s (Figure 1). He underwent an urgent insertion of a pacemaker, and IV methylprednisolone was escalated to 500 mg IV OD. His neurological symptoms deteriorated, including his inability to swallow, and he was, therefore, commenced on IVIG and 0.5 mg intramuscular neostigmine TDS. The patient was deemed not to be suitable for ICU and not for intubation and ventilation due to his frailty and comorbidities. Unfortunately, the patient's health continued to deteriorate, requiring 15 litres of high-flow oxygen to maintain oxygen saturations above 90%, and he subsequently passed away, two days after being admitted to hospital.

## 3. Discussion

We report four cases of overlap syndrome, with two proving to be fatal, and to our knowledge the first case report of overlap syndrome resulting from avelumab immunotherapy. It is estimated that ICI-related myocarditis occurs in approximately 0.27% to 1.14% of cases, ICI-related MG in 1–5%, and ICI-related myositis in 1% [8]. Myocarditis has the highest mortality of approximately 25–50%; however, with the presence of all three toxicities, the mortality rises significantly to 62.5% [1, 2]. Overlap syndrome can occur after a single dose of immunotherapy, as was the case for three of our reported patients.

Currently, there are no validated biomarkers to predict irAEs. Combining an ICI with another ICI or other anticancer treatment such as tyrosine kinase inhibitor, radiation or chemotherapy appears to be the predominant risk factor for developing cardiovascular irAEs [9]. The presence of preexisting autoimmune diseases increases the risk of developing irAEs, in particular ICI-related myositis and MG [10].

The underlying mechanism of overlap syndrome remains unclear. ICI-induced T-cell hyperactivation leading to tissue infiltration has been demonstrated in myocardial tissue. Additionally, ICIs deplete regulatory T cells, thereby activating anergic self-reactive T cells. There appears to be an expansion of T cell clonal cells in both tumour and muscle cells leading to a role of molecular mimicry [1, 5]. There is an increased incidence of MG in anti-PD1 therapy compared to anti-CTLA-4 therapy. This is thought to be related to the proliferation of autoreactive B cells creating antibodies to self-antigens on the AChR and neuromuscular junction as a result of PD-1 blockade on B cells [3]. Further research is required to establish the mechanisms of ICI-related overlap syndrome.

Myocarditis can present with a variety of symptoms including chest pain, dyspnoea, palpitations, and fatigue. American Society of Clinical Oncology (ASCO) suggests the spectrum ranges from asymptomatic, but with abnormal cardiac biomarkers or ECG, to life-threatening decompensation with end-organ damage [11]. Patients may present with arrhythmias, AV block, as in patients 1 and 4, tachycardia, or T-wave inversion, as in patient 3. Diagnostic testing is important to confirm the diagnosis and rule out other common cardiac causes such as acute coronary syndrome (ACS). This is especially important given that there is significant overlap in the risk factors for developing cancer and ACS. Measuring troponin, B‐type natriuretic peptide (BNP) and N‐terminal pro‐B‐type natriuretic peptide (NT‐proBNP), and inflammatory markers are the most commonly requested diagnostic blood markers. Troponin-I over troponin-T is recommended in ICI-related myocarditis as troponin-T may also be elevated in myositis [1]. In all our cases, troponin levels were significantly elevated. Mahmood et al. reported elevated troponin in 94% of cases with ICI-related myocarditis, elevated BNP and NT‐proBNP in 66%, and abnormal ECG in 89% [12]. An echocardiogram is useful in the diagnosis of myocarditis and may show reduced left ventricular ejection fraction or regional wall abnormalities. A baseline echocardiogram before commencing immunotherapy would, therefore, be useful to allow evaluation of any changes. European Society of Medical Oncology (ESMO) recommends a cardiovascular magnetic resonance (CMR) scan if troponin levels or left ventricular ejection fraction on echocardiogram is normal. If CMR is not diagnostic or not available, then a PET-CT/MRI is recommended. Should the diagnosis remain uncertain, an endomyocardial biopsy should be undertaken [8]. It is important to note, however, that CMR, PET, and endomyocardial biopsy may not be readily available in all centres. ESMO recommends treatment involves withholding ICIs and prompts initiation of 500–1000 mg/day of IV methylprednisolone for three days followed by escalation to 1000 mg/day and commencing second line immunosuppressants such as MMF in severe cases or conversion to 1 mg/kg/day of oral prednisolone in uncomplicated cases. ASCO recommends commencing prednisolone 1-2 mg/kg initially and escalating to 1000 mg methylprednisolone with secondary immunosuppressants in patients without immediate response [11]. It is important to consider the risk of MG exacerbation with high-dose corticosteroids, and so a multidisciplinary team approach is important.

Myositis has a broad range of symptoms including myalgia, proximal limb weakness, pain, and myasthenic symptoms such as ptosis. Importantly, myositis can be life-threatening, particularly if respiratory muscles are affected. It can often mimic MG, and so, it is important to differentiate the two through investigations. In all of our cases, CK was significantly elevated; however, there have been cases of CK-negative ICI-related myositis [13]. ASCO recommends measuring CK, transaminases, and aldolase, as these can all be elevated [14]. Interestingly, both patient 1 and patient 4 had raised transaminases which may have been due to myositis and not hepatitis as it was thought at the time, especially given bilirubin was normal for both. Myositis-specific antibodies are largely negative and not helpful in the diagnosis [2]. In diagnostic uncertainty, electromyography (EMG) and muscle biopsy can be helpful. The mainstay of treatment as recommended by both ESMO and ASCO, similar to myocarditis but at a lower dose, is the use of corticosteroids with oral prednisolone 0.5–1 mg/kg/day in grade 2 symptoms and in severe cases with cardiac or respiratory involvement, withholding ICIs and commencing 1-2 mg/kg/day IV methylprednisolone or higher doses as required. Consideration should be given to plasmapheresis, IVIG, and the use of immunosuppressants such as MMF [11].

MG is the most common neuromuscular joint related ICI toxicity. Patients may present with symptoms similar to that of myositis; however, the disease appears to be more severe in anti-PD-1-related MG with symptoms including facial weakness and bulbar symptoms being more common [15]. Diagnostic workup includes positive antibodies including anti-AChR, as in patients 2 and 4, and antimuscle-specific kinase (anti-MuSK). The rate of positive anti-AChR in ICI-related MG is reported to be 66.7% and lower than in classical MG which is 80–90%, while anti-MuSK is much lower at 5% [16, 17]. EMG may be useful if antibodies are negative. Regular pulmonary function assessment with negative inspiratory force and FVC is crucial for recognition of respiratory failure and prompt initiation of noninvasive or invasive ventilation. Both ASCO and ESMO recommend withholding ICIs and giving corticosteroids and pyridostigmine. Specifically, ASCO recommends, for grade 2 toxicities, pyridostigmine 30 mg titrated up to 120 mg TDS and prednisolone 1–1.5 mg/kg/day. In grade 3 or 4 toxicities, IVIG or plasmapheresis should be considered [11]. It is important to consider that pyridostigmine may increase the risk of conduction defects, and hence, a multidisciplinary approach is important [18].

## 4. Conclusion

We describe four cases of overlap syndrome and the first with the administration of avelumab. Overlap syndrome is associated with significant mortality and morbidity, and it is important that if clinicians suspect any of these irAEs, the others should also be considered. We recommend early involvement of specialty teams to manage these complex patients. Prompt recognition and early intervention are needed to treat these potentially life-threatening conditions. Further studies are needed to identify which patients might be more at risk, the pathophysiology, potential early biomarkers, and the optimal management approach.

## Figures and Tables

**Table 1:** Blood results for patient 1 showing an improvement after commencing methylprednisolone.

	Day 1	Day 2	Day 3	Day 4	Day 5	Day 6	Day 7	Day 8	Day 9	Day 11
Troponin (ng/L)	1051	1464	2105	1101	3875	3871	2312	2378	1218	228
ALT (U/L)	415	474			707	565	558	494	480	308
CK (IU/L)	11150	13028	13591	6713	3936	2794	1846	1230	1005	807

On day 5, the patient was transferred to a specialist centre, MMF was commenced, and methylprednisolone increased to 1000 mg OD.
